# Functional characterization of MEKK3 in the intestinal immune response to bacterial challenges in grass carp (*Ctenopharyngodon idella*)

**DOI:** 10.3389/fimmu.2022.981995

**Published:** 2022-08-05

**Authors:** Fufa Qu, Xuan Zeng, Zhenzhen Liu, Meixing Guo, Xia Zhang, Shenping Cao, Yonghua Zhou, Zhimin He, Jianzhou Tang, Zhuangwen Mao, Yalin Yang, Zhigang Zhou, Zhen Liu

**Affiliations:** ^1^ Hunan Provincial Key Laboratory of Nutrition and Quality Control of Aquatic Animals, Department of Biological and Environmental Engineering, Changsha University, Changsha, China; ^2^ Key Laboratory for Feed Biotechnology of the Ministry of Agriculture, Feed Research Institute, Chinese Academy of Agricultural Sciences, Beijing, China

**Keywords:** grass carp, MEKK3, molecular characterization, bacterial challenge, intestinal immunity

## Abstract

Mitogen-activated protein kinase kinase kinase 3 (MEKK3) is an evolutionarily conserved Ser/Thr protein kinase of the MEKK family that is essential for the host immune response to pathogen challenges in mammals. However, the immune function of MEKK3s in lower vertebrate species, especially in bony fish, remains largely unknown. In this study, a fish MEKK3 (designated *Ci*MEKK3) gene was cloned and identified from grass carp (*Ctenopharyngodon idella*). The present *Ci*MEKK3 cDNA encoded a 620 amino acid polypeptide containing a conserved S-TKc domain and a typical PB1 domain. Several potential immune-related transcription factor-binding sites, including activating protein 1 (AP-1), nuclear factor kappa B (NF-κB) and signal transducer and activator of downstream transcription 3 (STAT3), were observed in the 5’ upstream DNA sequence of *Ci*MEKK3. A phylogenetic tree showed that *Ci*MEKK3 exhibits a close evolutionary relationship with MEKK3s from *Cyprinus carpio* and *Carassius auratus*. Quantitative real-time PCR analysis revealed that *Ci*MEKK3 transcripts were widely distributed in all selected tissues of healthy grass carp, with a relatively high levels observed in the gill, head kidney and intestine. Upon *in vitro* challenge with bacterial pathogens (*Aeromonas hydrophila* and *Aeromonas veronii*) and pathogen-associated molecular patterns (PAMPs) (lipopolysaccharide (LPS), peptidoglycan (PGN), L-Ala-γ-D-Glu-mDAP (Tri-DAP) and muramyl dipeptide (MDP)), the expression levels of *Ci*MEKK3 in the intestinal cells of grass carp were shown to be significantly upregulated in a time-dependent manner. *In vivo* injection experiments revealed that *Ci*MEKK3 transcripts were significantly induced by MDP challenge in the intestine; however, these effects could be inhibited by the nutritional dipeptides carnosine and Ala-Gln. Moreover, subcellular localization analysis and luciferase reporter assays indicated that *Ci*MEKK3 could act as a cytoplasmic signal-transducing activator involved in the regulation of NF-κB and MAPK/AP-1 signaling cascades in HEK293T cells. Taken together, these findings strongly suggest that *Ci*MEKK3 plays vital roles in the intestinal immune response to bacterial challenges, which will aid in understanding the pathogenesis of inflammatory bowel disease in bony fish.

## Introduction

Mitogen-activated protein kinases (MAPKs) are a class of conserved serine and threonine protein kinases that are widely present in a variety of organisms and can participate in mediating multiple biological processes in response to extracellular stimuli and cellular stress (1, 2). MAPKs are activated through three-tiered kinase cascades: MAP kinase kinase kinase (MAP3K or MEKK), MAP kinase kinase (MAP2K or MEK) and MAPK (3, 4). In the MAPK signal cascade system, MEKKs are first activated by endogenous and environmental stimuli, which in turn activate downstream dual-specific MKKs that can further phosphorylate Thr and Tyr within the motif Thr-Xaa-Tyr of various MAPKs, including extracellular signal-regulated kinases (ERKs), c-jun N-terminal or stress-activated protein kinases (JNKs/SAPKs) and p38 MAPK, and finally induce the activation of various transcription factors that regulate the expression of effector genes participating in inflammation, apoptosis and development (4–7).

As the initial kinases of three-tiered kinase cascades, MEKKs are essential for signal transduction in the MAPK pathway and mainly consist of MEKK1, MEKK2, MEKK3, MEKK4, transforming growth factor-β-activating kinase 1 (TAK1), apoptosis signal-regulating kinase 1 (ASK1), dual leucine zipper bearing kinase (DLK), and tumor progression locus-2 (Tpl2) (8, 9). Among MEKK family members, MEKK3 has been shown to be highly conserved among eukaryotes and involved in the regulation of cell proliferation, the inflammatory response and tumor development (10–13). In mammals, MEKK3 has been reported to contain an activation loop (A-loop) domain, a Phox and Bem1p (PB1) domain and a serine/threonine kinase catalytic (S-TKc) domain (14). Previously, the A-loop domain was shown to contain some specific serine/threonine phosphorylation sites that are responsible for MEKK3 activation and signal transduction (15–18). The PB1 domain is a secondary structure rich in basic amino acids that could be involved in the transmission of specific intracellular signals in various signaling pathways by the PB1-PB1 interaction with other signal proteins (19–21). The S-TKc domain at the C-terminus of the MEKK3 protein has been shown to be conserved in MEKK family members and was critical for its activation and phosphorylation (22).

In mammals, MEKK3s have been shown to act as crucial regulators of innate immunity against pathogen infections *via* the involvement of interleukin-1 receptor (IL-1R) and toll-like receptor (TLR) -mediated NF-κB, JNK and p38 cascades, which are essential for inducing the expression of proinflammatory cytokines (12, 23–25). For example, Huang et al. reported that MEKK3 played a decisive role in IL-1-induced and LPS-induced interleukin (IL-6) production by regulating the IKK-NF-κB and JNK-p38 MAPK pathways in mouse embryonic fibroblast (MEF) cell lines (23). In MEKK3-deficient fibroblast cells, MEKK3 is essential for IKK activation and functions downstream of TNF receptor-associated factor 2 (TRAF2) and receptor-interacting protein (RIP) in the TNF-induced NF-κB pathway (24). A study of the macrophage line Raw264.7 showed that the production of LPS-induced IL-6 and granulocyte-macrophage colony-stimulating factor (GM-CSF) was significantly decreased in MEKK3 knockdown cells; however, this decrease was restored by reintroducing human MEKK3 cDNA (12). Recently, Li and colleagues demonstrated that LPS-stimulated proinflammatory cytokine (IL-1β, TNF-α and IL-6) production is significantly regulated by the TAK1-MEKK3 axis in myeloid cells (25). Using knockdown experiments in BV2 cells, it was found that MEKK3 plays a critical role in the development of neuroinflammation in Parkinson’s disease by regulating the NF-κB signaling pathway (26). A recent study in mice found that overexpression of MEKK3 significantly activated IRF7 to trigger strong induction of type I IFNs, while knockdown of MEKK3 *in vivo* substantially impaired type I IFN induction and increased susceptibility to HSV-1 infection (27).

Due to its important immunological function in mammals, MEKK3 in bony fish has also attracted much attention in recent years. For example, many MEKK3 genes have been cloned and identified in a variety of fish species such as *Danio rerio*, *Cyprinus carpio*, *Carassius auratus*, *Pimephales promelas* and *Triplophysa tibetana*. A recent study of a hybrid snakehead (*Channa maculate* ♀ × *Channa argus* ♂) showed that fish MEKK3 is involved in the innate immune response to *Nocardia seriolae* and *Aeromonas schubertii* challenges (28). However, compared with mammals, the immune function of MEKK3 in fish is still largely unclear. Grass carp (*Ctenopharyngodon idella*) is one of the most highly produced and economically important freshwater fish species in China. Over the past decades, bacterial enteritis has seriously harmed the healthy breeding of grass carp and caused serious economic losses to aquaculture (29–31). Investigation into the intestinal immune function of *C. idella* MEKK3, an essential signal transducer of the innate immune response, might facilitate the development of disease control and prevention measures. To this end, a fish MEKK3 (*Ci*MEKK3) gene was identified from *C. idella*, and its intestinal expression in response to bacterial pathogens (*Aeromonas hydrophila* and *Aeromonas veronii*) and PAMPs (lipopolysaccharide (LPS), peptidoglycan (PGN), L-Ala-γ-D-Glu-meso-diaminopimelic acid (Tri-DAP) and muramyl dipeptide (MDP)) challenges was investigated by using quantitative real-time PCR (qRT-PCR). In addition, *Ci*MEKK3 was overexpressed in human embryonic kidney 293T (HEK293T) cells to determine its intracellular localization characteristics and signal transduction function. The data from this study may help to illuminate the function of MEKK3s in the intestinal immunity of bony fish.

## Materials and methods

### Experimental animals, bacterial challenge and tissue collection

Healthy *C. idella* weighing approximately 30 g were obtained from Hunan Institute of Aquatic Science in Changsha, China, and acclimatized in 30 L tanks with circulating freshwater at 24 ± 1°C for two weeks prior to experimentation. Eight tissues including head kidney, spleen, intestine, liver, gill, blood, muscle and heart were collected from healthy individuals using sterilized scissors and tweezers for further tissue distribution analysis. Tissue samples were ground into powder in liquid nitrogen and stored at -80°C until RNA extraction.

For the *in vitro* immune challenge experiments, the cultured primary intestinal cells were challenged with bacterial pathogens (*A. hydrophila* and *A. veronii*) and PAMPs (LPS, PGN, Tri-DAP and MDP). The experimental protocol was performed according to our previous study (32). Before the challenge experiment, the *C. idella* intestinal cells were grown in 6-well culture plates with 2 mL of Dulbecco’s modified Eagle’s medium (DMEM, Gibco-BRL, USA) containing 10% fetal bovine serum (FBS, Gibco BRL, USA) per well at 28°C in a humidified incubator provided with 5% CO_2_. The two bacterial pathogen strains (*A. hydrophila* and *A. veronii*) were kindly provided by the Feed Research Institute, Chinese Academy of Agricultural Sciences (33), and cultured in LB medium at 37°C overnight for the challenge experiment. The *C. idella* intestinal cells were challenged with *A. hydrophila* (1×10^7^ cfu/mL), *A. veronii* (1×10^7^ cfu/mL), Tri-DAP (50 μg/mL; *In vivo*Gen), MDP (50 μg/mL; *In vivo*Gen), LPS (10 μg/mL; Sigma-Aldrich) or PGN (10 μg/mL; Sigma-Aldrich) and collected at 0, 3, 6, 12 and 24 h post-challenge. The cells were treated with phosphate-buffered saline (PBS, pH 7.4) used as a control. All the collected cell samples were placed in -80°C immediately and used for gene expression assays.

For the *in vivo* immune challenge experiments, healthy grass carp were injected with the bacterial dipeptide MDP and nutritional dipeptides (carnosine and Ala-Gln) using a 1 mL syringe. In the first immune challenge experiment, *C. idella* were randomly divided into two groups, the MDP challenge group and the control group, and each group was placed in separate tanks. The fish in the immune challenge group were injected with 100 μl MDP (10 μg/mL, *In vivo*Gen, USA). The control individuals were injected with an equal volume of PBS. After treatment, the grass carp were returned to the tanks and intestines of three individuals in each group were randomly sampled at 0, 3, 6, 12, 24, 48 and 72 h post-injection. In the second immune challenge experiment, grass carp were randomly divided into four groups and were injected intraperitoneally with 100 μl of PBS, MDP (10 μg/mL), MDP (10 μg/mL) + carnosine (5 mmol/L; Sigma-Aldrich) or MDP (10 μg/mL) + Ala-Gln (5 mmol/L; Sigma-Aldrich). Intestines were collected from three individuals in each tank at 12 and 24 h post-injection for gene expression level analysis.

All experiments were performed according to the recommendations of the Guidance of the Care and Use of Laboratory Animals in China. The research presented in this manuscript was approved by the Animal Ethics Committee of Changsha University.

### Total RNA isolation and cDNA synthesis

Total RNA was extracted from the harvested intestinal cells and adult tissues of grass carp using RNAiso Plus (Takara, Japan) reagent following the manufacture’s protocol. RNA concentration was measured using the ratio of UV absorbance at 260/280 nm in a NanoDrop 2000 spectrophotometer (Thermo Fisher, USA) and the quality was assessed using 1.5% agarose electrophoresis. The RNA samples were treated with gDNA Eraser (TaKaRa, Japan) to eliminate genomic DNA contamination. Total RNA from each sample was reverse transcribed using the PrimeScript™ 1st Strand cDNA Synthesis Kit (Takara, Japan) and PrimeScript™ RT Reagent Kit with gDNA Eraser (TaKaRa, Japan) to synthesize the cDNA template for gene cloning and expression analysis, respectively. Finally, the cDNA mix was diluted 10-fold and stored at -80°C for subsequent processing.

### Cloning the cDNA sequence of *Ci*MEKK3

Based on the reported *C. carpio* MEKK3 sequence from the GenBank database, gene-specific primers were designed to amplify the cDNA sequence of *Ci*MEKK3 by reverse transcription PCR (RT-PCR). PCR amplification was performed in a total reaction volume of 50 μl containing 1 μl of cDNA template, 37.75 μl of dH_2_O, 4 μl of dNTP mixture (2.5 mM each), 5 μl 10×Ex Taq Buffer (Mg^2+^ plus), 1 μl of each primer (10 μM) and 0.25 μl of Ex Taq DNA Polymerase (TaKaRa, Japan). The PCR conditions were as follows: 94°C for 3 min, 35 cycles of 94°C for 30 s, 57°C for 30 s, 72°C for 2 min, and 72°C for 10 min. The PCR products were separated using 1.2% agarose gel/TAE electrophoresis and then purified with a TaKaRa Agarose Gel DNA Purification Kit Ver.2.0 (TaKaRa, Japan). After purification, all of the specific PCR products were cloned into the pMD19-T vector (TaKaRa, Japan). The ligation product was transformed into *Escherichia coli DH5α*, and three positive colonies were screened and sequenced on a 3730 Applied Biosystems (ABI) DNA sequencer.

### Bioinformatics analysis

The nucleotide and deduced amino acid sequences of the cloned *Ci*MEKK3 gene were analyzed using the BLAST tool available from the National Center for Biotechnology Information (NCBI) (http://blast.ncbi.nlm.nih.gov/Blast.cgi). The molecular weight and theoretical isoelectric point were calculated using the pI/Mw tool (https://web.expasy.org/protparam/). The functional domains were deduced with the Simple Modular Architecture Research Tool (SMART) website (http://smart.embl-heidelberg.de/). Potential transcription factor binding sites (TFBSs) in the promoter region of *Ci*MEKK3 were predicted using JASPAR (http://jaspardev.genereg.net/) and AliBaba2 (http://gene-regulation.com/pub/programs/alibaba2/index.html). The exon-intron arrangement of *Ci*MEKK3 based on the DNA sequence from grass carp genome (http://www.ncgr.ac.cn/grasscarp/ ) was determined using the Spidey tool (http://www.ncbi.nlm.nih.gov/spidey/). The identity and similarity of amino acid sequences were calculated with MatGAT2.02 software. The alignment of multiple sequences of MEKK3s from different species was conducted using the MegAlign program with the Clustal W method, and GeneDoc software was employed to visualize the results. A neighbor-joining (NJ) phylogenetic tree of MEKK3 was constructed based on the amino acid sequences of MEKK3s using MEGA 5.0 software, with the bootstrap value set at 1000 replicates. The GenBank accession numbers for the various MEKK3s are as follows: [*Homo sapiens*] AAB41729.1, [*Papio anubis*] XP_017806347.1, [*Aotus nancymaae*] XP_021529429.1, [*Cebus imitator*] XP_017404666.1, [*Mus musculus*] NP_036077.1, [*Balaenoptera musculus*] XP_036692416.1, [*Danio rerio*] XP_688694.2, [*Triplophysa tibetana*] KAA0705702.1, [*Pimephales promelas*] XP_039542275.1, [*Cyprinus carpio*] XP_018936269.1, [*Carassius auratus*] XP_026062650.1, [*Ctenopharyngodon idella*] ON082069.

### Quantitative real-time PCR (qRT-PCR) analysis

Quantitative real-time PCR (qRT-PCR) was used to detect the relative mRNA expression levels of *Ci*MEKK3 with β-actin as an internal reference gene. Gene specific primers were designed based on the cDNA sequences of grass carp MEKK3 and β-actin using Primer Premier 5.0 software and are listed in **Table 1**. qRT-PCR was performed on a Quant-Studio™ 3 Real-Time PCR System (Thermo Fisher, USA) in a total volume of 16 μl containing 8 μl of 2 × SYBR Premix Ex Taq II (Tli RNaseH Plus) (Takara, Japan),1 μl of cDNA template, 0.32 μl of ROX, 0.64 μl of each primer, and 5.4 μl of nuclease-free water. The qRT-PCR procedure was as follows: 95°C for 5 min, followed by 45 amplification cycles of 10 s at 95°C, 30 s at 58°C, and 72°C for 10 s, and three biological replicates for each group were conducted. The specificity of each qRT-PCR product was confirmed by melting curve and agarose gel analysis. The relative expression levels of *Ci*MEKK3 were normalized to β-actin expression, and the relative expression values were calculated using the 2^-ΔΔCT^ method (34).

**Table 1:** Sequences of designed primers used in this study.

Primer	Sequence (5’ to 3’)	Comment
*Ci*MEKK3-F1	ACTTCAATCAATAGCACTCAC	CDS Cloning
*Ci*MEKK3-R1	TCCAGGCAACAGCTGATTGGGT	
*Ci*MEKK3-F2	CTGCGTGAACAGGGCGACTTG	Real-Time PCR
*Ci*MEKK3-R2	GGAGGGGAGGCATTGCTTTGT	
*Ci*β-actin-F	CTTGACTTCGAGCAGGAG	Real-Time PCR
*Ci*β-actin-R	GGCATACAGGTCTTTACGG	
*Ci*MEKK3-F3	GATAAGAGCCCGGGCGGATCCATGAATGAGAGACAG	*Ci*MEKK3-Flag
*Ci*MEKK3-R3	ATCGAATTCCTGCAGAAGCTTTCAGCACAAGATCTG	
*Ci*MKK4-F	GATAAGAGCCCGGGCGGATCCATGGCGACGTCCAGC	*Ci*MKK4-Flag
*Ci*MKK4-R	ATCGAATTCCTGCAGAAGCTTTCAGTCCACGTACAT	
*Ci*MKK6-F	GATAAGAGCCCGGGCGGATCCATGGAAGGAGGGAG	*Ci*MKK6-Flag
*Ci*MKK6-R	ATCGAATTCCTGCAGAAGCTTTCAGTCCCCAAGGAT	
*Ci*MKK7-F	GATAAGAGCCCGGGCGGATCCATGTCGTCGCTGGAG	*Ci*MKK7-Flag
*Ci*MKK7-R	ATCGAATTCCTGCAGAAGCTTCTACCTGCTGAAGAG	
*Ci*MEKK3-F4	CTACCGGACTCAGATCTCGAGATGAATGAGAGACAG	*Ci*MEKK3-GFP
*Ci*MEKK3-R4	ATGGTGGCGACCGGTGGATCCCGGCACAAGATCTGAG	

### Plasmid construction

The eukaryotic expression vectors pCMV-N-Flag*-Ci*MEKK3 (*Ci*MEKK3-Flag) and pEGFP-N1-*Ci*MEKK3 (*Ci*MEKK3-GFP) were constructed for mammalian cell transfections using the ClonExpress^®^ II One Step Cloning kit (Vazyme, China) according to the manufacturer’s protocol. The expression plasmids *Ci*MKK4-Flag, *Ci*MKK6-Flag and *Ci*MKK7-Flag were constructed for our previous studies (35, 36). The primer pairs (**Table 1**) were designed for amplification of the complete open reading frame (ORF) encoding the polypeptide of *Ci*MEKK3. The ORF of *Ci*MEKK3 was cloned into the *BamH* I/*Hind* III site of pCMV-N-Flag and *Xho* I/*BamH* I site of pEGFP-N1 to generate the plasmids *Ci*MEKK3-Flag and *Ci*MEKK3-GFP, respectively, and then transformed into *E. coli*, *DH5α* (TaKaRa, Japan). The colonies were screened on LB plates containing kanamycin at 37°C and sequenced for further verification. All transfection plasmids were prepared from overnight bacterial cultures using the HiPure Plasmid EF Mini Kit (Magen, China) according to the manufacturer’s instructions.

### Cell culture and transfection

Human embryonic kidney 293T (HEK293T) cells were cultured with DMEM (Gibco-BRL, USA) containing 10% FBS (Gibco BRL, USA) and antibiotics (100 mg/L streptomycin and 10^5^ U/L penicillin, Gibco) at 37°C in a 5% CO_2_ atmosphere. For plasmid-liposome transfection, cells were seeded overnight and grown to 80–90% confluence at the time of transfection. Then, plasmids were transfected into the cells using Lipofectamine 2000 (Invitrogen, USA) according to the manufacturer′s instructions.

### Subcellular localization

To investigate the subcellular localization of *Ci*MEKK3, 1 μg/well of the recombinant plasmid *Ci*MEKK3-GFP or empty plasmid pEGFP-N1 (control) was transfected into HEK293T cells. Prior to transfection, the cells were seeded on sterile coverslips at 1 × 10^5^ cells/well in 6-well plates for overnight growth. Then the cells were transfected using Lipofectamine 2000 with a 2:1 ratio of transfection reagent to endo-free plasmids in serum-free culture medium. At 48 h post-transfection, the transfected cells were fixed with 4% paraformaldehyde, washed with PBS and stained with DAPI to distinguish localization between the cytoplasm and nuclei. The subcellular localization results were observed under a fluorescence microscope.

### Dual-luciferase reporter gene assay

For the dual-luciferase reporter assays, cells were transiently cotransfected with luciferase reporter vectors NF-κB-Luc (Promega, USA)/AP-1-Luc (Promega, USA), pRL-TK (Promega, USA) (20 ng/well), and expression plasmids *Ci*MEKK3-Flag/*Ci*MKK4-Flag/*Ci*MKK6-Flag/*Ci*MKK7-Flag using Lipofectamine 2000 (Invitrogen, USA). The pRL-TK plasmid was used as the internal control and the plasmid pCMV-N-Flag was used as the negative control. Each experiment was performed in triplicate under similar conditions to obtain biological replicates. After the cells were transfected for 48 h, the firefly and Renilla luciferase activities were measured using a dual-Luciferase reporter assay system (Promega, USA) following the manufacturer’s instructions. The relative luciferase activity is presented as the ratio of firefly luciferase to renilla luciferase.

### Statistical analysis

All the data derived from luciferase assays and qRT-PCR were subjected to one-way analysis of variance (ANOVA) followed by LSD or Duncan’s *post-hoc* test to determine significant differences among the treatments. The results are shown as the means ± standard error of measurement (SEM). Differences were considered statistically significant at *P* < 0.05 and extremely significant at *P* < 0.01.

## Results

### cDNA cloning and sequence analysis of *Ci*MEKK3

**Figure 1:**
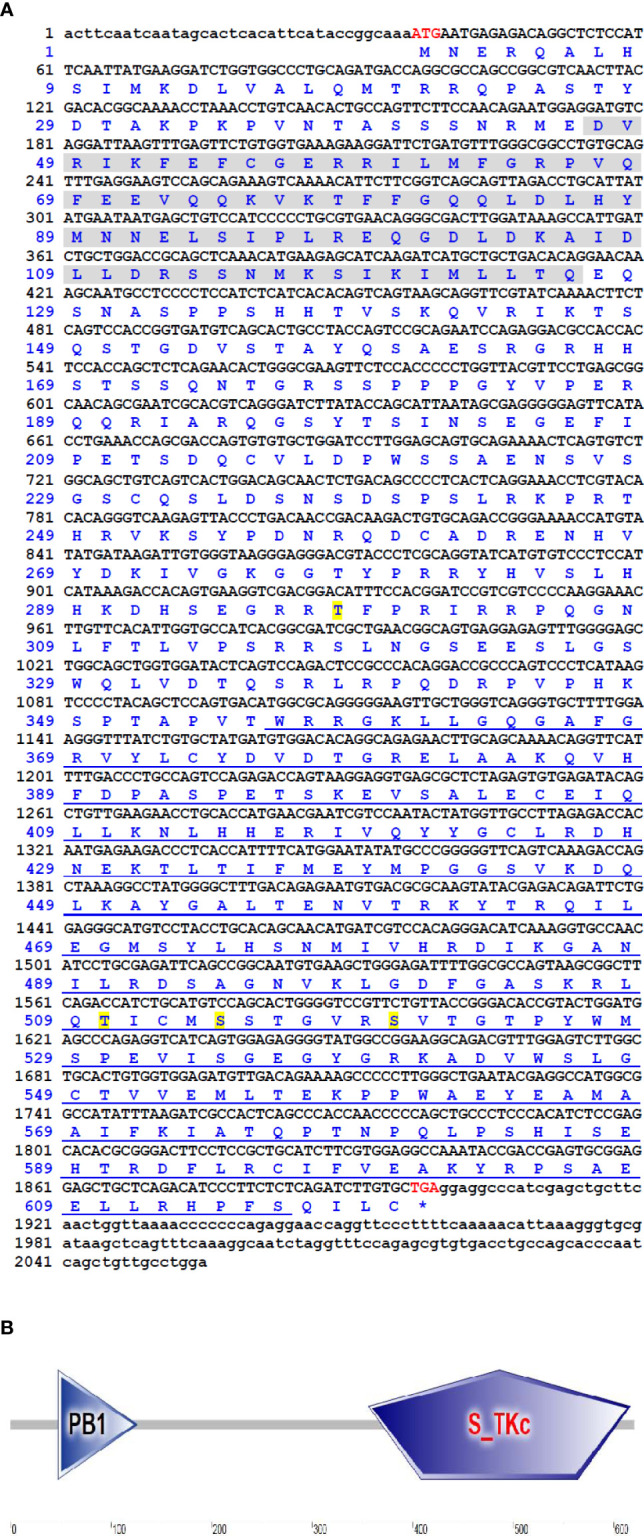
The cDNA sequence and deduced amino acid sequences of *Ci*MEKK3. **(A)** Nucleotides and amino acids are numbered on the left of the sequences. The start codon (ATG) and stop codon (TGA) are shown in red font. The ORF sequence of *Ci*MEKK3 is indicated in uppercase letters, while the 5′- and 3′-UTR sequences are shown in lowercase. The PB1 domain and S_TKc domain are marked by gray shading and blue underline, respectively. The predicted phosphorylation sites are shown by yellow shading. **(B)** Functional domains of *Ci*MEKK3 were predicted using the SMART tool.

**Figure 2:**
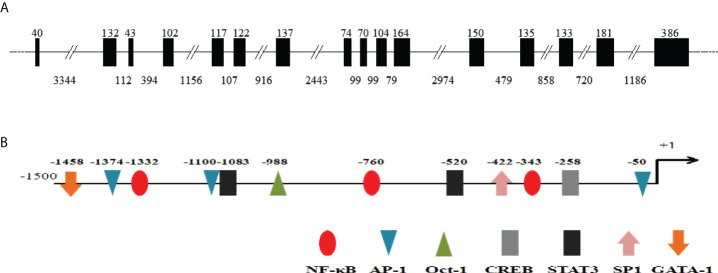
Genomic organization and 5′ flanking regions of *Ci*MEKK3. **(A)** The intron-exon organization of *Ci*MEKK3. Exons and introns are indicated by black boxes and black lines with the corresponding sizes (bp), respectively. **(B)** Transcription factor binding sites in the 5′ flanking regions (~1.5 kb) of *Ci*MEKK3. The predicted binding sites are shown by different colors of ellipses, triangles, arrows or rectangles.

The cDNA sequence of *Ci*MEKK3 was obtained with RT-PCR and submitted to the GenBank database (No. ON082069). The *Ci*MEKK3 cDNA was 2055 bp in length, which included a 5′-untranslated region (UTR) of 36 bp, a 3′-UTR of 156 bp, and an ORF of 1863 bp encoding a 620 amino acid residue (**Figure 1A**). The predicted molecular weight of *Ci*MEKK3 was 70.17 kDa, and the theoretical pI was 9.15. Similar to other orthologs, several phosphorylation sites such as Thr294, Thr516, Ser520 and Ser526 were observed in the amino acid residue of *Ci*MEKK3 (**Figure 1A**). Structural analysis based on the SMART program revealed that MEKK3 contained two conserved domains, including a PB1 domain (positions 47–126 aa) and a typical S_TKc domain (positions 356–616 aa), with the typical features of MEKK3 family proteins (**Figure 1B**). The genomic organization of *Ci*MEKK3 was analyzed by comparing the genomic DNA and cDNA sequences. As shown in **Figure 2A**, the DNA sequence of *Ci*MEKK3 possesses a multiexonic gene structure containing sixteen exons separated by fifteen introns, and its mature mRNA sequence was generated by appropriate splicing. Using the JASPAR and AliBaba2 programs, several transcription factor-binding sites, including three nuclear factor kappa B (NF-κB) sites, three activating protein 1 (AP-1) sites, one octamer-binding transcription factor 1 (Oct-1) site, one cAMP response element-binding protein (CREB) site, two signal transducer and activator of downstream transcription 3 (STAT3) sites, one specificity protein 1 (SP1) site and a GATA-binding factor 1 (GATA-1) site were found in the 5’-upstream DNA sequence of *Ci*MEKK3 (**Figure 2B**).

### Multiple sequence alignment and phylogenetic analysis

**Figure 3:**
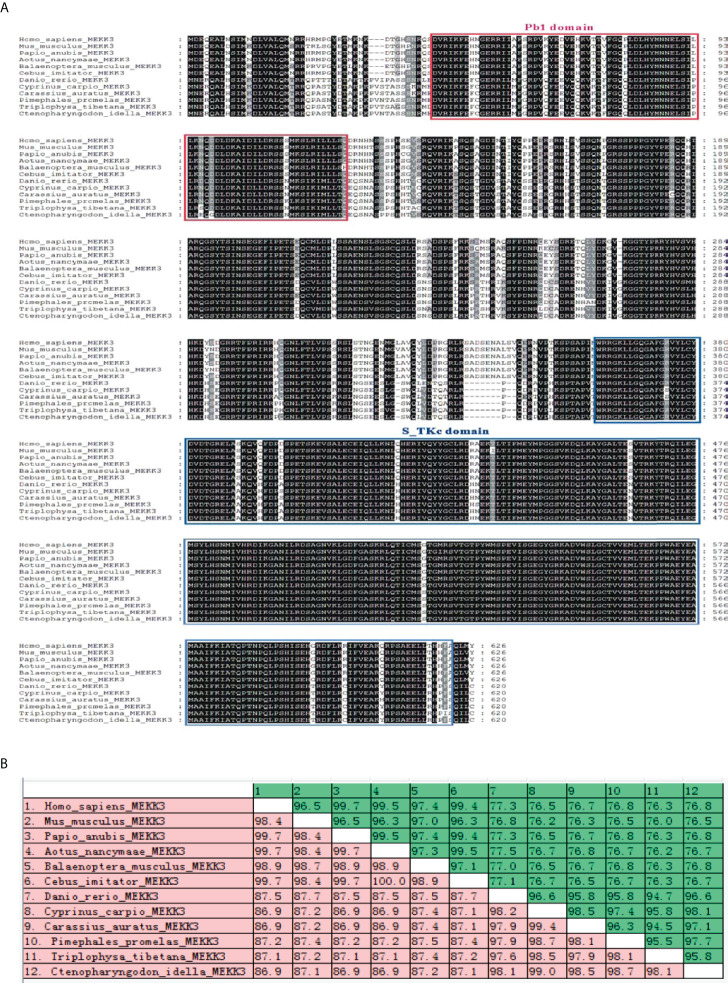
Multiple alignment of *Ci*MEKK3 with other reported MEKK3 from GenBank database. **(A)** Identical amino acids are shaded in black and similar amino acids are shaded in gray. The conserved PB1 domain and S_TKc domain are indicated by the red box and blue box, respectively. **(B)** The similarities (red) and identities (green) of amino acid sequences were analyzed using MatGAT2.02 software.

**Figure 4:**
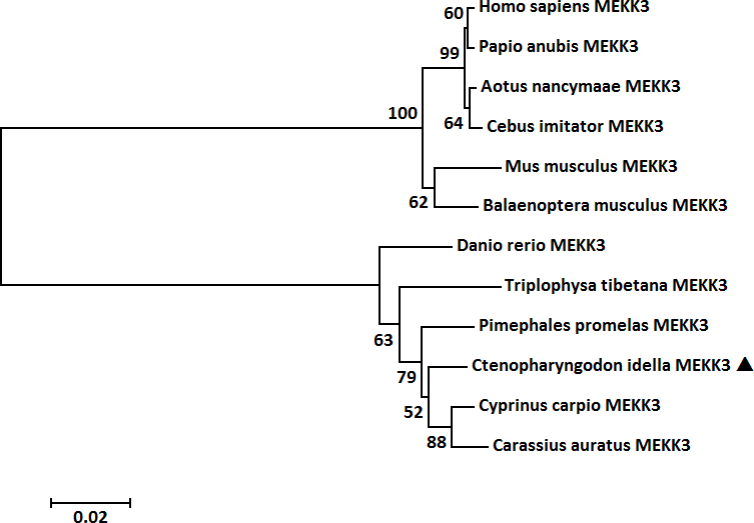
Phylogenetic tree of MEKK3 proteins from representative vertebrate species. The tree was constructed with the maximum likelihood method in MEGA 5.0 software with 1000 bootstrap replications. The bar (0.02) indicates the genetic distance. *Ci*MEKK3 is shown by a black triangle.

Multiple sequence alignment illustrated that the amino acid sequence and functional domains were conserved in vertebrate MEKK3 counterparts and that all contained a typical S-TKc domain and a PB1 domain (**Figure 3A**). A MatGAT2.01 analysis was conducted to generate a measure of similarity and identity for the *Ci*MEKK3 protein with other homologs. The deduced amino acid sequence of *Ci*MEKK3 shared 76.5–98.1% identity (I) and 86.9–99.0% similarity (S) with MEKK3 sequences from other vertebrate species. Among all the selected MEKK3 sequences, *Ci*MEKK3 was closest to that of *Cyprinus carpio* (98.1% I, 99.0% S), followed by that of *Pimephales promelas* (97.7% I, 98.7% S) (**Figure 3B**). To investigate the evolutionary relationships of MEKK3s, a phylogenetic tree was constructed using the MEKK3 sequences of twelve representative vertebrate species. Overall, the relationships displayed in the cladogram generally agree with those of traditional taxonomy. The MEKK3s from mammals and fish were clustered separately into two branches, and *Ci*MEKK3 was embedded within the fish cluster. In addition, *Ci*MEKK3 exhibited a close evolutionary relationship with MEKK3s from *Cyprinus carpio* and *Carassius auratus* (**Figure 4**).

### Tissue expression pattern and subcellular localization of *Ci*MEKK3

**Figure 5:**
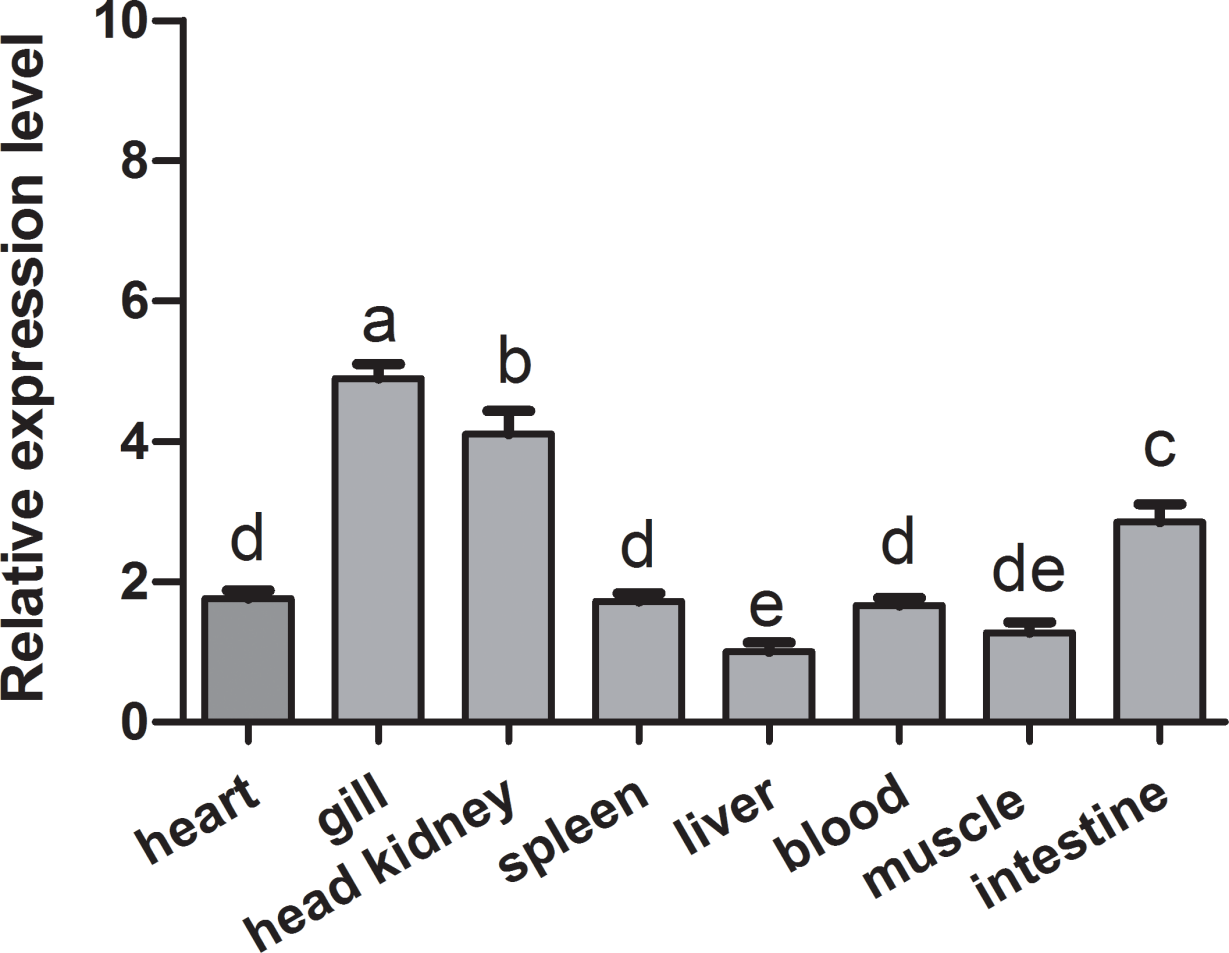
Relative expression levels of *Ci*MEKK3 in various tissues of healthy grass carp. Each bar represents the mean of the normalized expression levels of the replicates (*N* = 3). Bars marked with different letters indicate significant differences among different tissues (*P* < 0.05).

**Figure 6:**
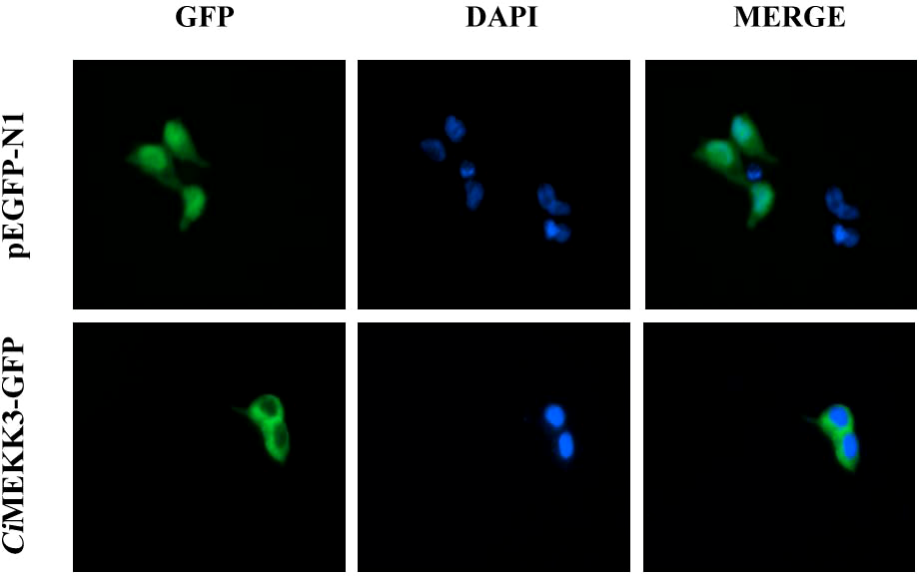
Subcellular localization of *Ci*MEKK3 in HEK293T cells. HEK293T cells were transfected with pEGFP-N1 (upper row) or *Ci*MEKK3-GFP (lower row). At 48 h post-transfection, the cells were fixed with 4% paraformaldehyde, stained with 4′,6-diamidino-2-phenylindole (DAPI), and then observed with fluorescence microscopy.

The qRT-PCR analysis was employed to determine the tissue expression profiles of *Ci*MEKK3 in healthy grass carp. *Ci*MEKK3 was ubiquitously expressed in all eight examined organs (intestine, liver, blood, muscle, heart, gill, head kidney and spleen), with the highest expression levels in the gill, followed by the head kidney and intestine, and relatively low expression levels in the liver (**Figure 5**). To obtain the subcellular localization characteristics of *Ci*MEKK3, HEK293T cells were transfected with plasmid pEGFP-N1 or *Ci*MEKK3-GFP using Lipofectamine 2000. Based on the results obtained from fluorescence microscope, *Ci*MEKK3 was distributed mainly in the cytoplasm while the control protein was dispersed throughout the cytoplasm and the nuclear areas, suggesting that the *Ci*MEKK3 protein may be a cytoplasm-localized protein in HEK293T cells (**Figure 6**).

### Time-dependent expression of *Ci*MEKK3 in intestinal cells in response to pathogen challenge

**Figure 7:**
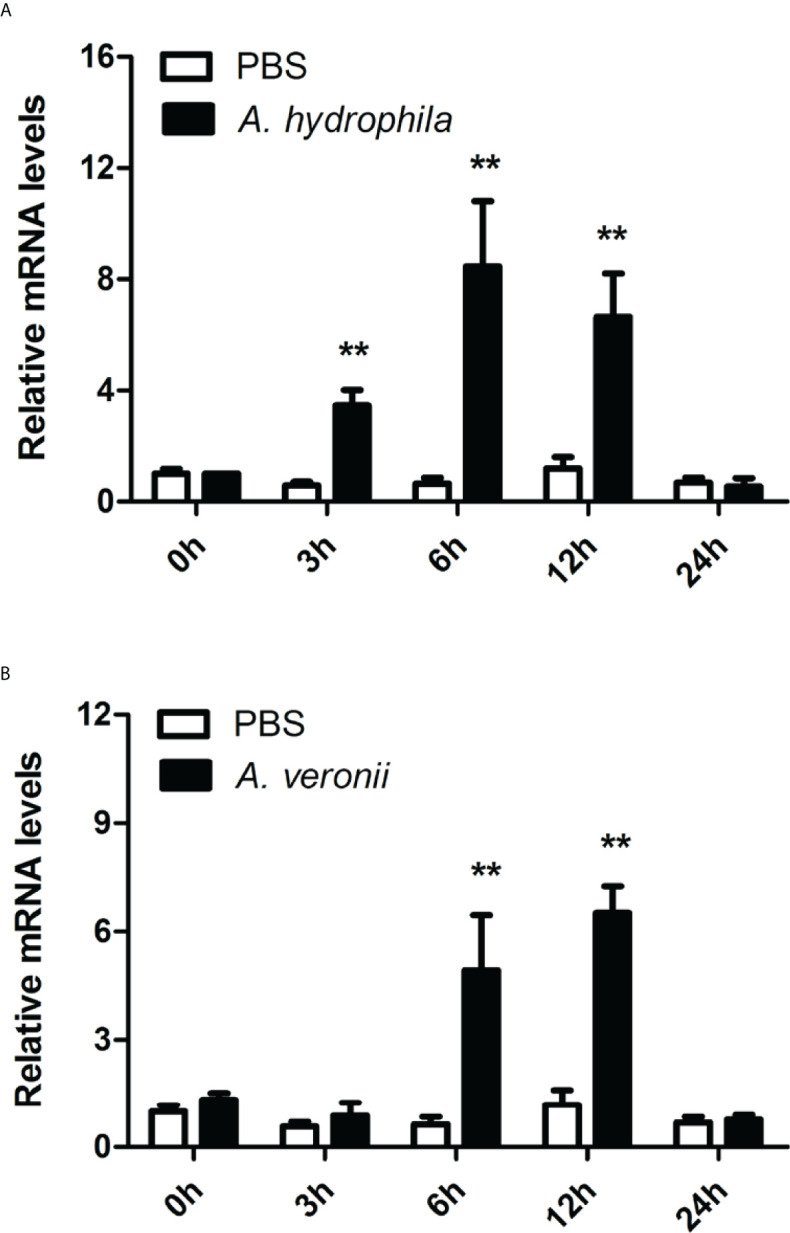
Temporal expression profiles of *Ci*MEKK3 mRNA in intestinal cells after challenge with *A. hydrophila* **(A)** or *A veronii* **(B)**. Each bar represents the mean of the normalized expression levels of replicates (*N* = 3). Significant differences between the challenge group and the control group are indicated with an asterisk (** represents *P* < 0.01).

The transcriptional responses of *Ci*MEKK3 were monitored in intestinal cells after stimulation with *A. hydrophila* and *A. veronii*. The qRT-PCR results showed that *Ci*MEKK3 exhibited a strong and elevated response to *A. hydrophila* and *A. veronii* infection, and its expression levels in intestinal cells were significantly regulated by these two bacterial pathogen challenges (**Figure 7**). When challenged with *A. hydrophila*, the mRNA levels of intestinal *Ci*MEKK3 were upregulated at 3 h post-stimulation (*P* < 0.01), reaching a peak value at 6 h post-stimulation *(P *< 0.01), and then returning to control levels at 24 h post-stimulation (**Figure 7A**). Upon infection with *A. veronii*, *Ci*MEKK3 transcripts in intestinal cells did not significantly increase until 6 h post-stimulation (*P* < 0.01), reached the highest level at 12 h post-stimulation (*P* < 0.01), and then sharply decreased to the original level at 24 h post-stimulation (**Figure 7B**).

### Time-dependent expression of *Ci*MEKK3 in intestinal cells in response to PAMP challenge

**Figure 8:**
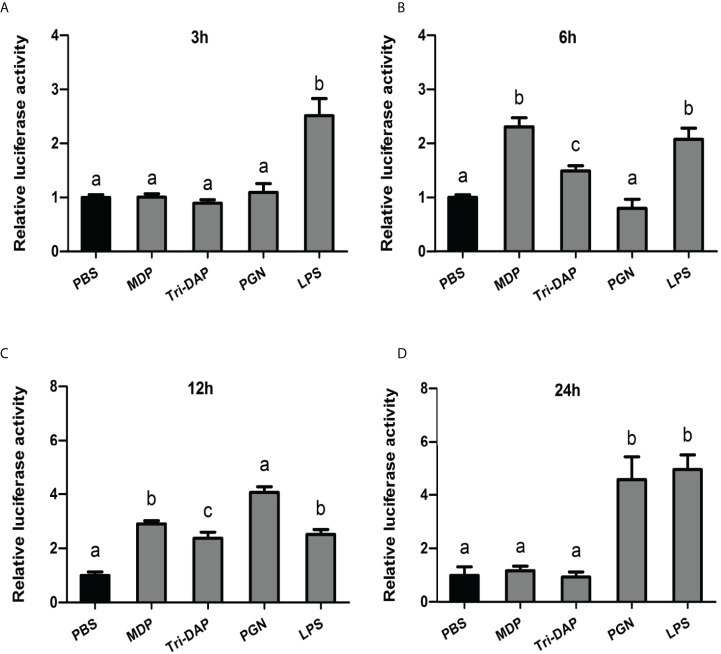
Temporal expression profiles of *Ci*MEKK3 mRNA in intestinal cells at 3 h **(A)**, 6 h **(B)**, 12 h **(C)** and 24 h **(D)** post-challenge with MDP, Tri-DAP, PGN or LPS. Data are shown as the mean ± standard error of three individual fish (*N* = 3). Significant differences between the challenge group and the control group are indicated with different letters (*P* < 0.05).

To further investigate the immune function of *Ci*MEKK3 in intestinal cells *in vitro*, the expression levels of *Ci*MEKK3 were detected after stimulation with typical bacterial PAMPs (MDP, Tri-DAP, PGN and LPS) *via* qRT-PCR (**Figure 8**). In the first 3 h of the immune challenge, *Ci*MEKK3 expression only significantly increased in the LPS group compared with that of the PBS control (*P* < 0.05) (**Figure 8A**). After 6 h of stimulation, the transcript levels of *Ci*MEKK3 were shown to be significantly induced by MDP, Tri-DAP and LPS (*P* < 0.05) (**Figure 8B**). Interestingly, all selected bacterial PAMPs (MDP, Tri-DAP, PGN and LPS) significantly upregulated the expression levels of *Ci*MEKK3 at 12 h post-stimulation (*P* < 0.05) (**Figure 8C**). Upon 24 h of PAMP challenge, *Ci*MEKK3 expression was maintained at a relatively high level in the LPS and PGN groups (*P* < 0.05) but returned to control levels in the MDP and Tri-DAP groups (*P* > 0.05) (**Figure 8D**).

### Time-dependent expression of *Ci*MEKK3 in intestines in response to MDP challenge

**Figure 9:**
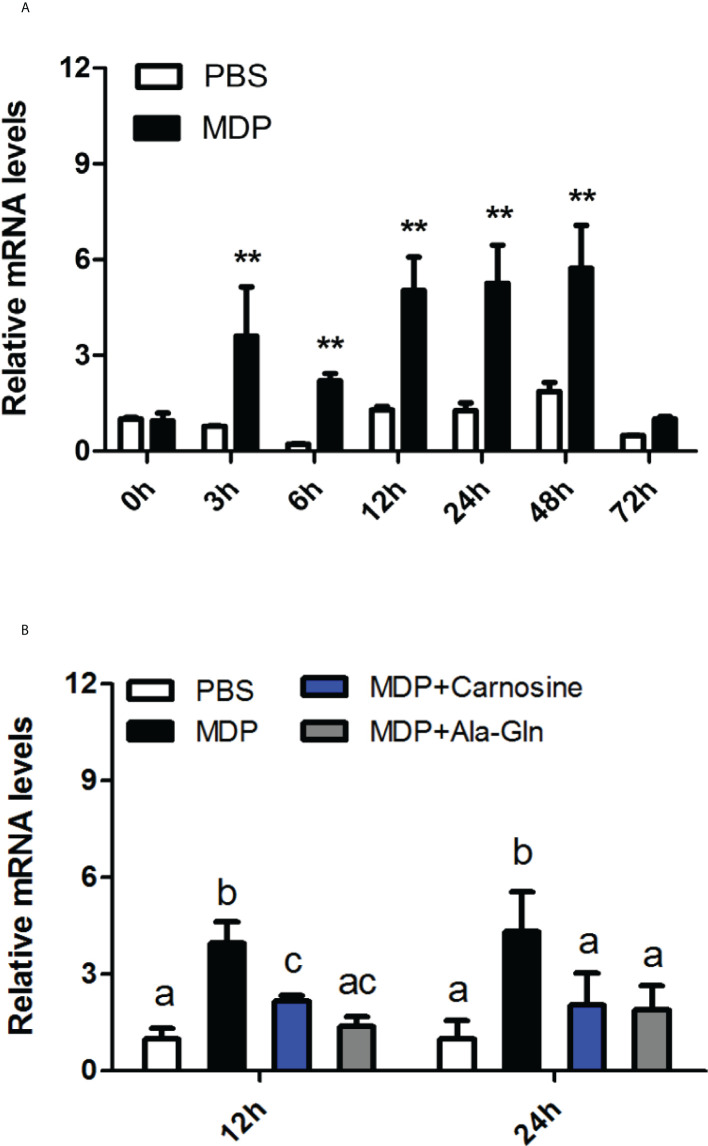
Temporal expression profiles of *Ci*MEKK3 mRNA in the intestine after injection with MDP **(A)** or MDP + carnosine/Ala-Gln **(B)**. Comparative analysis and statistical tests were performed on the challenge groups and PBS group at the same time point. Each bar represents the mean of the normalized expression levels of replicates (*N* = 3). Significant differences are indicated with an asterisk (** represents *P* < 0.01) or different letters (*P* < 0.05).

The time-course expression levels of the *Ci*MEKK3 transcripts were detected in the MDP-injected intestines of grass carp *in vivo*. As shown in **Figure 9A**, the relative expression of *Ci*MEKK3 mRNA was significantly increased at 3 h, 6 h, 12 h, 24 h and 48 h post-injection (*P* < 0.05) and sharply decreased at 72 h post-injection (*P* > 0.05) in comparison with the control group. Additionally, a nutritional dipeptide (carnosine or Ala-Gln) and MDP were coinjected into grass carp to study the regulatory mechanism underlying the bacterial MDP-induced expression of *Ci*MEKK3 in intestine. The results from **Figure 9B** show that the inductive effect of MDP on *Ci*MEKK3 expression was significantly inhibited by carnosine or Ala-Gln treatment in the intestine of grass carp. These data may imply that the nutritional dipeptides carnosine and Ala-Gln may act as effective regulators to alleviate the bacterial MDP-mediated intestinal inflammatory response.

### Effects of *Ci*MEKK3 overexpression on the NF-κB and AP-1 signaling pathways

**Figure 10:**
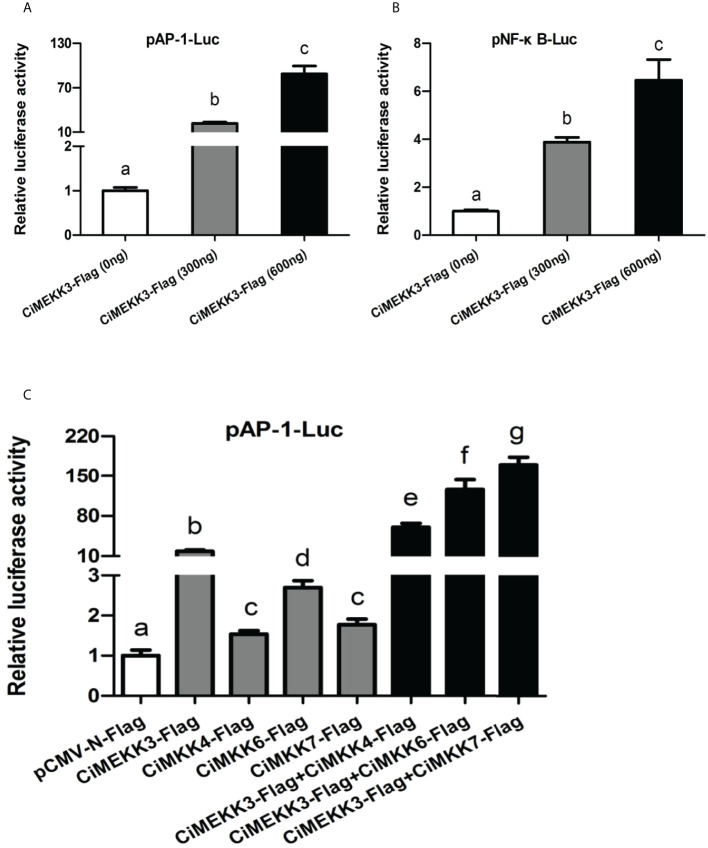
Effects of *Ci*MEKK3 overexpression on the activity of the AP-1 and NF-κB pathways. The *Ci*MEKK3-Flag (0, 300 and 600 ng/well) was cotransfected with 100 ng/well AP-1-Luc **(A)** or NF-κB-Luc **(B)** into HEK293T cells. **(C)** *Ci*MEKK3-Flag (300 ng/well) was cotransfected with 100 ng/well AP-1-Luc, 300 ng/well *Ci*MKK4-Flag, *Ci*MKK6-Flag or *Ci*MKK7-Flag into HEK293T cells. Each bar represents the mean (three replicates) ± standard deviation. Firefly and Renilla luciferase activities were detected in cell lysates 48 h after transfection. Data are the fold changes relative to the empty vector transfected cells. Significant differences are indicated with different letters (*P* < 0.05).

Dual-luciferase reporter assays were performed to determine the possible role of *Ci*MEKK3 in the NF-κB and AP-1 signaling pathways. The NF-κB and AP-1 luciferase reporter were significantly activated by overexpression of *Ci*MEKK3 in a dose-dependent manner in HEK293T cells (**Figure 10A**, **B**). In particular, it was found that the activating effects of *Ci*MEKK3 overexpression on the AP-1 pathway were stronger than those on NF-κB signaling. Additionally, the luciferase reporter results showed that the activation effects on the AP-1 luciferase reporter of cells cotransfected *Ci*MEKK3-Flag with *Ci*MKK4-Flag, *Ci*MKK6-Flag or *Ci*MKK7-Flag were significantly higher than those of cells transfected with *Ci*MEKK3 or *Ci*MKKs alone (**Figure 10C**), suggesting that *Ci*MEKK3 could enhance the downstream MKK-induced activation of the AP-1 signaling pathway. Collectively, the present results clearly indicate that *Ci*MEKK3 might serve as an effective activator of the NF-κB and AP-1 signaling pathways.

## Discussion

In vertebrates, MEKKs are essential signaling molecules of the NF-κB and MAPK pathways which play important regulatory roles in the immune response to pathogenic challenges (37, 38). To date, several MEKK family members have been identified from fish such as TAK1 in *Oncorhynchus mykiss* (39), *Paralichthys olivaceus* (40) and *Megalobrama amblycephala* (41), c-Raf in *Epinephelus coioides* (42) and *Oreochromis niloticus* (43), and MAP3K4 in *C. idella* (32). However, information regarding MEKK homologs in bony fish remains limited. In the present study, a member of the fish MEKK family, *Ci*MEKK3, was cloned from *C. idella* using RT-PCR technology. Similar to other reported MEKK3 proteins, the present *Ci*MEKK3 contains a conserved S-TKc domain and PB1 domain, which were shown to be essential for its activation and interaction with other signaling molecules in the MAPK pathway (19–22). In addition, the kinase catalytic domain of *Ci*MEKK3 contains several phosphorylation sites (Thr294, Thr516, Ser520 and Ser526), which were observed in MEKK3s from mammals. Previous studies have demonstrated that the phosphorylation of specific Ser/Thr sites directly affects MEKK3 activation and its function in intracellular signal transduction under physiological and pathological conditions (15–18). These findings suggested that MEKK3s may possess similar phosphorylation mechanisms and signal transduction functions in mammals and fish. Based on the analysis of the 5′-upstream DNA sequence, several potential immune-related transcription factor-binding sites including NF-κB, AP-1, CREB and STAT3 were observed in the promoter region of *Ci*MEKK3, suggesting that it may be involved in immune-related processes in grass carp. Multiple sequence alignment analysis showed that *Ci*MEKK3 shares higher identity and similarity with other fish MEKK3s than with the reported mammalian homologs. A phylogenetic tree based on the amino acid sequences of MEKK3s revealed that *Ci*MEKK3 shares a close relationship with *C. carpio* and *C. auratus* MEKK3. These results indicated that *Ci*MEKK3 is a novel member of the fish MEKK3 family.

Previous studies have reported that MEKK3 is ubiquitously expressed in various tissues and cell types in mammals (12, 14, 25, 44). Recently, fish MEKK3 has been shown to be constitutively expressed in all tissues of healthy hybrid snakehead, including the liver, spleen, head kidney, trunk kidney, skin, gill, muscle, intestine, heart, brain, and blood (28). Similarly, the broad expression patterns of other MEKK family members including TAK1 (39, 40) and MEKK4 (32) have also been observed in fish. In our study, tissue expression analysis revealed that *Ci*MEKK3 is broadly expressed in all selected tissues of healthy grass carp, consistent with the tissue-expression profile of other reported MEKK3s, suggesting the potential roles of *Ci*MEKK3 in various biological processes. In bony fish, the head kidney and intestine are important immune-related tissues that are essential for host defense responses to immune challenges (29, 45). It is well known that fish gills are in direct contact with the aquatic environment, which serves as the first line of immune defense against various pathogen infections (46). Our qRT-PCR results show that *Ci*MEKK3 displays relatively higher expression levels in the gill, head kidney and intestine, indicating that *Ci*MEKK3 may play potential roles in the innate immunity of grass carp. To further explore the distribution and function of *Ci*MEKK3, the subcellular localization of the *Ci*MEKK3 protein was examined using a fluorescence microscope. The results reveal that *Ci*MEKK3 is distributed mainly in the cytoplasm, suggesting that MEKK3 may act as a cytoplasmic localized protein involved in the signal transduction process of the MAPK pathway. Similar results have been observed in hybrid snakehead MEKK3 where *Cc*MEKK3 is exclusively distributed in the cytoplasm of HEK293T cells (28). Moreover, it was noted that other fish MEKK family members, including TAK1 (47), c-Raf (42) and MEKK4 (32), also exist in the cytoplasm of the cells, implying that MEKKs may be mainly involved in biological events in the cytoplasm.

Bacterial enteritis is one of the most frequent and serious infectious diseases that occurs in the intensive cultivation of grass carp (29–31). However, the exact pathogenesis of bacterial enteritis is still not well understood. Similar to other bony fish, innate immunity has been considered a first line of host defense against invading pathogens in the intestines of grass carp (48). Over the past decades, several immune-related signaling pathways, including TLRs (49), IL-1R (50), NF-κB (51) and MAPK/AP-1 (52), have been identified in grass carp and proven to play important roles in the inflammatory response during pathogen infection. In mammals, MEKK3 was shown to act as an essential signal transducer of TLR- and IL-1R-mediated NF-κB, JNK and p38 cascades in response to immune challenge (23). MEKK3 was reported to be involved in regulating the LPS-induced production of proinflammatory cytokines in myeloid cells (25). Recently, a study of fish MEKK3 revealed its potential role in the immune response to pathogens and PAMPs challenge in the hybrid snakehead (28). To determine whether fish MEKK3 is involved in bacterial-induced intestinal inflammation, the expression levels of *Ci*MEKK3 were detected in response to typical aquatic pathogens (*A. hydrophila* and *A. veronii*) in intestinal cells of grass carp. Our results show that the transcript levels of *Ci*MEKK3 in intestinal cells are significantly induced by *A. hydrophila* and *A. veronii* challenges, suggesting that gram-negative bacterial strains can activate the intestinal MEKK3 pathway in grass carp. To gain more clues about the function of *Ci*MEKK3 in intestinal immune responses, we further analyzed the expression profile of *Ci*MEKK3 after stimulation with LPS and PGN, the important components of gram-negative and gram-positive bacteria, in intestinal cells at different time points. The qRT-PCR data indicated that *Ci*MEKK3 transcripts have a strong responsiveness to LPS and PGN challenges, further implying its potential role in intestinal defense against bacterial infections.

In recent years, much progress has been made with regard to the pathogenesis of bacterial-induced intestinal inflammation in mammals (53). Recent research has found that products of bacterial cell-wall PGN, including MDP and Tri-DAP, could be transported by peptide transporter 1 (PepT1) in epithelial cells of the small intestine, then recognized by the intracellular NBS-LRR proteins (NOD1 and NOD2) and finally result in the transcription of proinflammatory genes to initiate intestinal inflammation through a series of signaling events (54, 55). A large number of studies have shown that bacterial peptide MDP- and Tri-DAP-mediated PepT1/NOD signaling pathways are essential for the intestinal inflammatory response in mammals (56, 57). However, whether MDP and Tri-DAP can induce the intestinal inflammation and their related immune signaling pathways remains poorly understood in bony fish. Over the past few years, our laboratory has conducted work on bacterial peptide-mediated intestinal inflammation in grass carp. Our previous studies showed that the bacterial peptides MDP and Tri-DAP could induce intestinal inflammation and that MAPK pathways participated in the regulation of the intestinal immune response to bacterial peptide challenges in *C. idella* (52, 58). To better understand the regulatory mechanism of bacterial peptide-induced intestinal inflammation, the expression profile of *Ci*MEKK3 was analyzed after challenge with MDP and Tri-DAP in the intestine of grass carp. The *in vitro* experiment showed that *Ci*MEKK3 transcript levels in the intestine were significantly increased in a time-dependent manner upon MDP and Tri-DAP challenge. Moreover, we found that the intestinal expression levels of *Ci*MEKK3 induced by MDP challenge could be blocked by the nutritional peptides carnosine and Ala-Gln. Previously, it was reported that PepT1 ligand Lys-Pro-Val (KPV) could inhibit NF-кB signaling and decrease the production of proinflammatory cytokines in Caco2-BBE and Jurkat cells (59). These findings suggested that carnosine and Ala-Gln may exert an anti-inflammatory role similar to that of KPV, which may be useful for the future treatment of intestinal inflammation.

Nuclear factor-κB (NF-κB) is a group of immune-related transcription factors that play a predominant role in regulating the expression of various immune effectors, including proinflammatory cytokines, antimicrobial peptides and chemokines (60). MEKK3 has been previously shown to participate in the NF-κB signal transduction pathway (23, 24, 26). For example, MEKK3 has been shown to be essential for TNF-induced NF-κB activation in fibroblast cells (24). Additionally, Sun et al. reported that MEKK3 is a central intermediate signaling component in lysophosphatidic acid (LPA) -induced activation of NF-κB. In ovarian epithelial cells, overexpression of MEKK3 has been proven to increase NF-κB activity and the expression of Bcl-2, Bcl-xL and survivin (61). In addition to the NF-κB pathway, MEKK3 is involved in the regulation of MAPK/AP-1 signaling activation (62). Reportedly, MEKK3 is involved in TNFα, IL-1β, and TLR-induced MAPK activation *in vivo* and *in vitro* (23). To determine whether fish MEKK3 could activate the NF-κB and AP-1 signaling pathways, *Ci*MEKK3 expression plasmids were cotransfected with the AP-1 or NF-κB luciferase reporter genes into HEK293T cells. Our dual-luciferase reporter assays revealed that overexpression of *Ci*MEKK3 alone could significantly induce the activation of the AP-1 and NF-κB luciferase reporter, which was similar to the results observed in MEKK3 of hybrid snakehead (28). These results suggest that fish MEKK3 may also act as a positive regulator of the NF-κB and AP-1 signaling pathways. Moreover, our results showed that *Ci*MEKK3 may enhance the *Ci*MKK4-, *Ci*MKK6- and *Ci*MKK7-induced activation of the AP-1 luciferase reporter. MKK6 and MKK7 specifically phosphorylate and activate p38 and JNK, respectively, while MKK4 can act as an activator of both the p38 and JNK pathways (63, 64). These findings may suggest that *Ci*MEKK3 regulates the activity of the p38- and JNK-induced AP-1 signaling pathways by interacting with downstream MKKs. Combined with the gene expression profile during immune challenge, it is speculated that *Ci*MEKK3 may act as an important signal transducer of the NF-κB, JNK and p38 MAPK cascades involved in the intestinal immune response of grass carp. However, more experimental evidence is needed to support this speculation.

In conclusion, a functional fish MEKK3 gene (*Ci*MEKK3) was identified and characterized in grass carp, which contained the typical characteristic features of the MEKK3 family. Tissue-specific expression analysis showed that *Ci*MEKK3 mRNA was highly expressed in immune-related tissues of *C. idella*. The intestinal expression levels of *Ci*MEKK3 mRNA were significantly upregulated after challenge with bacterial pathogens (*A. hydrophila* and *A. veronii*) and PAMPs (MDP, Tri-DAP, PGN and LPS). Moreover, overexpression analysis revealed that *Ci*MEKK3 acted as an intracellular signaling molecule involved in the regulation of the NF-κB and AP-1 pathways in HEK293T cells. These results suggested that *Ci*MEKK3 plays essential roles in the intestinal immune response to bacterial challenges, which may provide new insights into the intestinal immunity of bony fish.

## Data availability statement

The datasets presented in this study can be found in online repositories. The names of the repository/repositories and accession number(s) can be found below: https://www.ncbi.nlm.nih.gov/, ON082069.

## Ethics statement

All experiments were performed according to the recommendations of the Guidance of the Care and Use of Laboratory Animals in China. The research presented in this manuscript was approved by the Animal Ethics Committee of Changsha University.

## Author contributions

FQ, ZL, and XZe designed the experiments and wrote the manuscript; XZe, ZZL, MG, and XZh conducted the experiments; XZe and ZZL analyzed the data; SC, YZ, ZH, JT, ZM, YY, and ZZ modified the manuscript; all authors reviewed and approved the final manuscript.

## Funding

This research was supported by the National Natural Science Foundation of China (Grant Nos. U21A20267, 32102813, 31902345 and 31702378), the Hunan Provincial Natural Science Foundation of China (Grant Nos. 2022JJ10053 and 2020JJ4642), the science and technology innovation Program of Hunan Province (2020RC3053), the Project of Scientific Research of the Hunan Provincial Education Department, China (Grant Nos. 20A045 and 20B058), the Training Program for Excellent Young Innovators of Changsha (Grant Nos. kq1707015, kq1802044, kq1905001, kq2009072 and kq2106067).

## Conflict of interest

The authors declare that the research was conducted in the absence of any commercial or financial relationships that could be construed as a potential conflict of interest.

## Publisher’s note

All claims expressed in this article are solely those of the authors and do not necessarily represent those of their affiliated organizations, or those of the publisher, the editors and the reviewers. Any product that may be evaluated in this article, or claim that may be made by its manufacturer, is not guaranteed or endorsed by the publisher.
